# Molecular explorations of cancer biology and therapeutics at PDB-101

**DOI:** 10.1038/s41388-022-02424-5

**Published:** 2022-08-10

**Authors:** David S. Goodsell, Shuchismita Dutta, Maria Voigt, Christine Zardecki, Stephen K. Burley

**Affiliations:** 1 Research Collaboratory for Structural Bioinformatics Protein Data Bank, Rutgers, The State University of New Jersey, Piscataway, NJ 08854 USA; 2 Institute for Quantitative Biomedicine, Rutgers, The State University of New Jersey, Piscataway, NJ 08854 USA; 3 Cancer Institute of New Jersey, Rutgers, The State University of New Jersey, New Brunswick, NJ 08901 USA; 4 Department of Integrative Structural and Computational Biology, The Scripps Research Institute, La Jolla, CA 92037 USA; 5 Research Collaboratory for Structural Bioinformatics Protein Data Bank, San Diego Supercomputer Center, University of California, La Jolla, CA 92093 USA; 6 Department of Chemistry and Chemical Biology, Rutgers, The State University of New Jersey, Piscataway, NJ 08854 USA

**Keywords:** Cancer, Molecular biology, Biochemistry

The Protein Data Bank (PDB) was established in 1971 as the first open-access digital data resource in biology [1]. It currently houses more than 190,000 three-dimensional (3D) structures of biological macromolecules (proteins, DNA, and RNA, and their complexes with one another and small molecule drugs, etc.), providing valuable insights into biochemical and biological function. Now in its 51st year of continuous operations, the PDB has become a leading global resource for experimental data central to discovery across fundamental biology, biomedicine, energy sciences, and bioengineering/biotechnology [2]. By providing open access to 3D structure data for the molecules of life, the PDB archive enables understanding of how normal cell growth is controlled, and how cancer cells bypass these essential controls to proliferate and metastasize. Open access to PDB data also facilitates discovery and development of novel anti-cancer agents, many of which are the product of structure-guided drug design using the tools of macromolecular crystallography [3].

PDB-101 (PDB101.rcsb.org) is an online web portal designed for educators, students, and the general public to promote exploration of the shapes and interactions of proteins and nucleic acids [4] (“101”, as in an entry-level course). It was established in 2011 by the RCSB Protein Data Bank [2, 5, 6], a global resource for advancing research and education in biology and medicine and the US data center for the Worldwide PDB partnership [7, 8].

Every two years, the RCSB PDB selects a biennial public health theme to focus PDB-101 content development and educator/student engagement. The theme for 2022 and 2023 explores the molecular mechanisms of *Cancer Biology and Therapeutics* and is reflected across the web portal.

The *Molecule of the Month* article series, launched by RCSB PDB in 2000, has introduced millions to the shapes and functions of 3D structures archived in the PDB. Each of the 270 installments freely available on PDB101.rcsb.org includes an introduction to the structure and function of a PDB molecule, discussion of the relevance of the molecule to human health and welfare, and suggestions as to how PDB-101 visitors can view these structures and access further details. While topical features such as Coronavirus and Opioid Receptors draw large audiences, articles related to the topics commonly addressed in classrooms (e.g., hemoglobin, catalase) continue to be heavily accessed year after year.

In January 2022, an intensive science communication “boot camp” was co-hosted virtually by the Rutgers Institute for Quantitative Biomedicine (IQB) and the RCSB PDB. Undergraduate and graduate students researched pre-selected topics in Cancer Biology and Therapeutics, including HER2/neu and Trastuzumab [9]; Nicotine, Cancer, and Addiction [10]; Non-Homologous End Joining Supercomplexes [11]; Pyruvate Kinase M2 and the Warburg Effect [12]; Secretory Antibodies [13]; and Vascular Endothelial Growth Factor (VegF) and Angiogenesis [14]. Boot camp participants hailed from the following colleges and universities around the United States: Fort Lewis College, CO; Hampton University, VA; Hunter College, NY; Nova Southeastern University, FL; Montclair State University, NJ; Pennsylvania State University, PA; Rutgers University, NJ; and University of Puerto Rico-Mayaguez, PR.

**Fig. 1:**
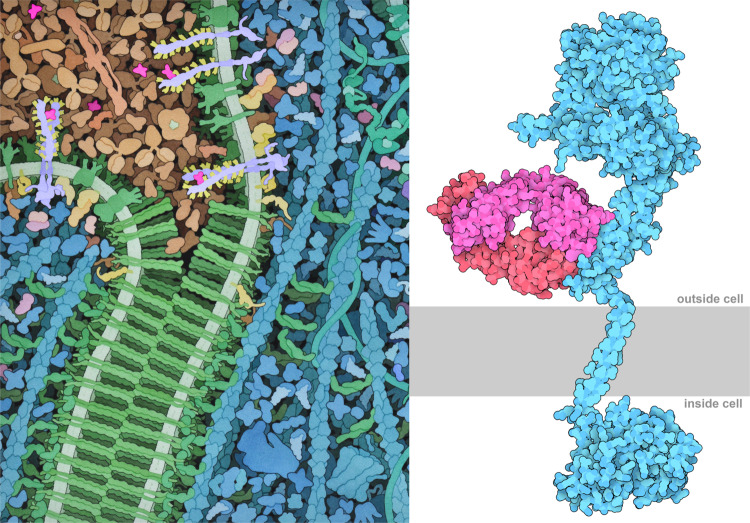
Images from cancer-focused Molecule of the Month articles. (left) Artistic conception of VegF signaling. VegF (magenta, top left) arrives at the potential site of a new blood vessel by traveling through the blood plasma (tan). VegF brings together two copies of VegFR (top center, lavender/yellow) to form an active dimer. Active VegFR then initiates a signal cascade that leads to intracellular phosphorylation of many proteins, including cadherin (green). The phosphorylated cadherins separate, making room for new blood vessels. Full image available at PDB-101 and in the article on Vascular Endothelial Growth Factor (VegF) and Angiogenesis [14]. (right) Trastuzumab (red/pink) antibody bound to HER2 (blue). The cell membrane is shown schematically in gray. The illustration is built from three PDB structures: the extracellular domain with the antigen-binding fragment (Fab) of trastuzumab (1n8z [15]), the kinase domain inside the cell (3pp0 [16]), and the transmembrane domain (2ks1 [17]). Image available from the article on HER2/neu and Trastuzumab [9].

For each of the Cancer-related *Molecule of the Month* articles, a team of four students identified exemplar structures from the PDB archive to tell the molecular story and then created stunning molecular visualizations (Fig. 1). Articles were co-authored under the guidance of Rutgers IQB faculty (coauthors DSG, SD, and SKB), and then externally reviewed for accuracy by biomedical researchers expert in the subject matter.

PDB-101 also hosts an annual Video Challenge for High School students (https://pdb101.rcsb.org/events/video-challenge/the-challenge). For the 2022 challenge, participating students were asked to explore two molecular signaling systems important in cancer: the p53/p21 pathway and the EGFR/Ras pathway. In all, 38 teams created short videos, each telling a coherent story explaining scientific concepts and touching on public health aspects of cancer, such as screening, prevention, and awareness. External expert reviewers selected the winning videos based on scientific content and communication effectiveness, while the public was invited to vote for a “Viewer’s Choice” video. Award winning entries can be found on the PDB-101 web portal at https://pdb101.rcsb.org/events/video-challenge/2022-awards.

All cancer-related educational materials available from PDB-101 can be found at https://pdb101.rcsb.org/browse/cancer. To support and provide context for these materials, PDB-101 also offers diverse educational curricula and activities to introduce young scientists to structural biology, comprehensive search and browse capabilities to help visitors find information, and a “Guide to Understanding PDB Data” to help visitors access and explore primary structural data stored in the PDB archive.
